# Supplementary material for: Are carbon emissions trading and green financial instruments synergistic? -Comprehensive quantitative research based on content analysis
Source: PLoS One. 2024 Mar 7;19(3):e0298601. doi: 10.1371/journal.pone.0298601 (PMC10919601; doi:10.1371/journal.pone.0298601)
Supplement: S1 Data — (ZIP) [file pone.0298601.s001.zip › Data/地级市Z_F工作报告.zip!/║╙─╧/╨φ▓2╩╨/└┤╫╘╣1⁄2╓┌║┼í╛┬φ┐╦╩2╛▌═°í┐_2018.docx]

各位代表：

现在，我代表市人民政府，向大会报告工作，请予审议，并请各位政协委员和其他列席人士提出意见。

一、2017年工作回顾

2017年是许昌发展进程中极不平凡的一年。在省委、省政府和市委的坚强领导下，市政府团结带领全市人民，以习近平新时代中国特色社会主义思想为指导，深入学习贯彻党的十九大精神，坚持稳中求进工作总基调，统筹稳增长、促改革、调结构、惠民生、防风险各项工作，圆满完成市七届人大一次会议确定的各项任务。全市实现生产总值2642.1亿元，增长8.7%，增速、总量分居全省第1位、第4位，提前3年比2010年翻一番；一般公共预算收入145.3亿元，增长14.2%；规模以上工业增加值1455.5亿元，增长9%；固定资产投资2531.8亿元，增长11.8%；社会消费品零售总额891.3亿元，增长12.2%；居民人均可支配收入21816元，增长9.8%。经济社会发展主要目标任务全面完成并好于预期。

（一）着力稳定经济运行，发展态势稳中向好。突出稳定工业增长，规模以上工业完成主营业务收入6662.2亿元，增长15.4%；实现利润559.3亿元，增长18.1%。着力扩大有效投资，180个重点项目完成投资1050亿元，生物医药产业园、泛在5G小镇等99个项目开工，平煤隆基高效单晶硅等35个项目竣工投产。落实降成本政策措施，减轻企业负担21亿元，一批企业渡过难关、恢复生机。着力防范化解风险，守住了不发生区域性系统性风险的底线。大力发展民营经济，主营业务收入超百亿元民营企业6家，4家入围“中国民营企业500强”，森源集团入选“中国企业500强”，民营经济占经济总量的80%以上，已成为许昌的亮丽名片。

（二）着力加快新旧动能转换，转型发展蹄疾步稳。打出“1+3+11”转型发展“组合拳”，加快11个重点产业转型升级，成为全省唯一转型发展观摩示范市。坚持优化存量与扩大增量并重，持续优化工业结构，制造业增加值占规模以上工业的95%，装备制造业增速保持20%左右，对规模以上工业贡献率达60%。加快发展现代服务业，服务业增加值增长10.3%；积极创建国家健康养老示范区和国家健康旅游目的地，成功举办首届鄢陵国际健康峰会；大力发展现代金融业，入选国家产融合作试点城市，“新三板”挂牌企业达25家，许昌农商银行挂牌开业，金融机构达117家。深化农业供给侧结构性改革，新建高标准粮田22万亩、高效节水灌溉面积19.3万亩，粮食产能稳定在280万吨左右，新型农业经营主体不断发展壮大。坚持质量立市、质量强市，蝉联全省政府质量工作考核第1名。

（三）着力推动区域协调发展，城乡建设提质增效。扎实推进新型城镇化，常住人口城镇化率突破50%，中心城区人口突破100万，城乡结构发生历史性变化。“郑许融合”升级为“郑许一体化”，“六个对接”持续深化，许港快速通道建成通车，公交化运营的郑许市域铁路工程许昌段开工建设，在积极融入郑州大都市区建设中抢占了先机、迈出了实质性步伐。统筹百城建设提质和文明城市创建，完成投资782亿元，城市中轴全线贯通，中央公园正式开园，曹魏古城（一期）、神垕古镇开街迎宾，许鄢快速通道等一批重大项目建成投用。加快棚户区改造，新开工5.08万套，基本建成3.88万套，超额完成省定任务。启动实施“四改一增”工程，成为全国老旧小区改造试点城市。广泛开展国土绿化，建设国家储备林基地13万亩。深入推进“五城联创”，是全省唯一的国家生态园林城市、中国十佳宜居宜业宜游城市和全省首个国家水生态文明城市，以全省第一名蝉联全国文明城市称号。加快组团城市发展，统筹中心镇和美丽乡村建设，完成440个中心村电网改造，新建改建农村道路576公里，新解决农村自来水人口45.2万人，在全省改善农村人居环境年度考核中位居第一。

（四）着力深化改革开放创新，发展活力动力持续增强。扎实推进供给侧结构性改革，认真落实“去降补”任务，关闭煤矿15处，化解煤炭过剩产能306万吨，胜利完成省定任务；提前完成剥离省属国有企业办社会职能和省定“僵尸企业”处置任务，被评为“全省国企改革攻坚战先进省辖市”。深化“放管服”改革，行政审批事项保持全省最少，事项办理时限平均压缩43%，不动产登记1天完成。加快商事制度改革，推行“三十五证合一”，新增各类市场主体5万户，增长16.7%。城管执法体制改革走在全省前列，其他重点领域和关键环节改革稳步推进。加大开放招商力度，新签约项目268个，到位资金582亿元；主动融入“一带一路”，对德合作取得新突破，落地项目11个、新签约项目25个、新增合作意向29个；加强开放平台建设，保税物流中心（B型）申建获省政府批复。强化科技创新，扎实推进“许昌英才计划”，认定表彰创新创业人才团队42个、高层次人才400名，新增省级以上创新平台38家，高新技术企业突破100家，专利申请量、授权量分居全省第2位、第3位，市城乡一体化示范区获批国家“双创”示范基地，科技进步对全市经济增长贡献率达60%。

（五）着力保障和改善民生，人民群众获得感不断提升。坚持以人民为中心的发展思想，扎实办好省定、市定民生实事，民生财政支出207.5亿元，占一般公共预算支出的72.4%。深入推进精准扶贫、精准脱贫，实现35个贫困村退出、2.1万人稳定脱贫。强力推进环境污染防治，空气优良天数、PM10、PM2.5指标均优于省定目标；3个省控河流出境断面达标率均为100%，是全省大气、水污染防治考核4个双优秀省辖市之一。稳定扩大社会就业，城镇新增就业7.6万人，农村劳动力新增转移就业7.9万人，返乡创业农民工1.47万人，城镇“零就业家庭”动态保持为零。完善社会保障体系，稳步提高城乡低保补助标准、企业退休人员养老金、机关事业人员退休金，城乡低保人员、残疾人、孤儿等困难群体生活保障更加充分。启动实施基础教育提升三年攻坚计划，新建成幼儿园42所、中小学78所，新增学位6.1万个，完成“全面改薄”学校396所。全面取消公立医院药品加成，扎实推进分级诊疗试点。加快卫生、体育、文化等公共事业发展，“15分钟便民服务圈”“15分钟医疗服务圈”“15分钟健身圈”“15分钟阅读圈”基本建成。新增集中供暖10万人。顺利通过国家食品安全示范城市中期评估。加强社会治理创新，深化平安许昌建设，抓好“三类突出问题”防范化解，安全生产形势平稳，社会大局和谐稳定。

各位代表！全市政府系统坚持以党的建设统领各项工作，严格落实全面从严治党主体责任，深入推进“两学一做”学习教育常态化制度化，认真落实中央八项规定及实施细则精神，持之以恒纠正“四风”，风清气正、干事创业的良好氛围更加浓厚。自觉接受人大监督、政协民主监督和社会监督，依法执行人大决议，认真办理人大代表建议和政协委员提案。加快法治政府建设，加强政府立法工作，完善政府法律顾问、“三重一大”决策等制度，坚持依法、民主、科学决策，政府治理能力不断提升。

持续扎紧制度笼子，扩大政务公开，强化审计监督，权力运行更加阳光透明。同时，积极推进军民融合发展，河南省（许昌）军民融合产业基地获批建设，“许昌舰”正式入列服役，驻许解放军和武警部队为地方发展作出新贡献，拥军优属、拥政爱民取得新成效。民族、宗教、外事、侨务、对台工作全面加强，妇女儿童、残疾人、慈善、红十字工作健康发展，统计、史志、档案、气象、农机、地震、人防、机关事务、援疆等工作取得新成绩。

各位代表！我们取得的发展成绩，是习近平新时代中国特色社会主义思想科学指导的结果，是省委、省政府和市委坚强领导的结果，是市人大、市政协监督支持的结果，是全市人民团结一心、开拓奋进的结果。在此，我代表市人民政府，向全市人民，向人大代表、政协委员，向驻许人民解放军指战员、武警官兵、公安民警致以崇高的敬意！向所有关心支持许昌发展的社会各界人士表示衷心的感谢！

安不忘危，兴不忘忧。我们清醒认识到，经济社会发展中还面临不少困难和问题：一是发展质量、创新能力有待进一步提升，新旧动能接续转换较慢；二是潜在风险依然较多，安全生产形势仍不乐观；三是民生欠账较多，脱贫攻坚和环境保护任务艰巨，教育、文化、卫生、养老等社会事业还有不少短板；四是政府职能转变还不到位，一些政府工作人员能力素质不适应新时代要求，一些领域不正之风和腐败问题还时有发生。对此，我们一定勇于担当，切实解决，不负人民重托。

二、2018年目标任务和重点工作

2018年是全面贯彻党的十九大精神的开局之年，是改革开放40周年，是决胜全面建成小康社会、实施“十三五”规划承上启下的关键一年。今年全市经济社会发展主要预期目标是：生产总值增长8%，规模以上工业增加值增长8.5%左右，服务业增加值增长9.5%，全社会固定资产投资增长9%左右，社会消费品零售总额增长10%，一般公共预算收入增长9%，税收占一般公共预算收入比重稳定在70%以上，进出口保持平稳增长，城镇调查失业率和城镇登记失业率分别控制在5%以内、4%以内，常住人口城镇化率提高1.6个百分点左右，研究与试验发展经费占生产总值的比重进一步提高，居民人均可支配收入增速高于经济增长速度，居民消费价格涨幅控制在3%左右；节能减排、环境保护、脱贫攻坚完成省下达任务。

今年以来，我们坚持以党的十九大精神和习近平总书记调研指导河南时的重要讲话为统领，坚持推动发展是第一要务、抓好党建是最大政绩，坚持稳中求进工作总基调，坚持新发展理念，坚持高质量发展要求，统筹推进“五位一体”总体布局和协调推进“四个全面”战略布局，以“四个着力”为根本遵循，以打好“四张牌”为总纲，以满足人民日益增长的美好生活需要为目标，聚焦打好“三大攻坚战”，统筹稳增长、促改革、调结构、惠民生、防风险，经济社会持续健康发展，高质量发展迈出了坚实步伐。特别是认真学习贯彻省委十届六次全会暨省委工作会议精神，以及市委七届六次全会精神，以党的建设高质量推动经济发展高质量，取得了一批在全省、全国具有一定影响的成绩。许昌市被确定为2018年国务院大督查和省政府集中督查“免督查”单位，均为全省唯一；全国促进民间投资经验交流现场会在许昌召开，国家发改委将许昌确定为促进民间投资工作联系点；中德（许昌）中小企业合作区成功获批，全省独此一家；成为全国地级市唯一的国家标准化改革创新先行区；全省改善农村人居环境工作会议在许昌召开，“许昌经验”推广全省；高质量发展成果受到省委、省政府充分肯定和中央、省级媒体集中报道；城市宜居度和居民幸福感、获得感、对生态环境的满意度4项指标均居全省第一，许昌实现更高质量发展的条件和优势更加凸显。

当前，我国经济运行面临一些新问题新挑战，外部环境发生明显变化，中央作出了“稳中有变”的总体判断和“六稳”的安排部署。我们将按照中央政治局会议、省委十届六次全会和市委七届六次全会的安排部署，牢牢把握稳中求进工作总基调，紧紧围绕高质量发展根本要求，持续抓好8个方面重点工作，确保圆满完成全年目标任务，努力在践行新发展理念上为全省探索路径、打造样板。

（一）坚决打好打赢“三大攻坚战”。切实肩负重大政治责任，抓重点、补短板、强弱项，为全面建成小康社会奠定坚实基础。防范化解重大风险方面，积极防控政府债务风险，化解存量，严控增量，建立健全政府债务动态监控和问责机制，坚决遏制变相举债和隐性负债。有效防范化解金融风险，严厉打击非法集资、非法互联网金融服务、恶意逃废银行债务等行为，持续优化金融生态环境。稳妥化解企业风险，因企制宜，分类管控，依法化解。有力防控社会风险，深化平安许昌建设，强化重点领域安全专项治理，推进突发事件预警信息发布体系建设，提升防灾减灾救灾能力；深入开展扫黑除恶专项斗争，始终保持高压态势，确保社会大局和谐稳定。精准脱贫方面，坚持以脱贫攻坚统揽经济社会发展全局，落实精准方略，提高脱贫质量，严格“两不愁三保障”标准，完成9个贫困村退出、1.75万贫困人口稳定脱贫任务。实施稳定增收计划，推进“千企帮千村”活动，大力发展扶贫产业，加快建设扶贫项目，拓宽贫困人口就业途径，促进贫困群众长期稳定增收。统筹抓好我市与淅川县、长葛市与桐柏县结对帮扶工作。污染防治方面，坚持长短结合、铁腕治污，实施环境治理提效行动，突出重点企业、区域、时段，落实“六控”，严治“两散”，确保圆满完成年度目标任务。加快实施水资源、水生态、水环境、水灾害统筹治理，严格落实河湖长制，全面消除建成区黑臭水体。深入实施土壤污染防治行动计划，加强土壤污染管控和修复。

（二）持续加快转型发展。以供给侧结构性改革为主线，全面落实“高端化、绿色化、智能化、融合化”要求，深入实施“1+3+11”转型发展方案，加快建设现代产业体系，推动经济高质量发展。大力发展先进制造业。坚持一手抓优化存量，加快实施绿色改造、智能改造、技术改造，推动电力装备、再生金属及制品等传统优势产业提档升级；坚持一手抓扩大增量，大力发展高纯硅电子材料、生物医药等新兴产业，积极培育新的经济增长点。提升现代服务业发展水平。积极发展健康养老产业，抓好“一院一校一中心”、康养小镇等重点项目建设，争创国家健康养老示范区。大力发展物流产业，加快建设保税物流中心（B型），推动物流业对外开放，打造中原地区重要的现代物流中心。支持鄢陵县争创国家级旅游度假区，加快神垕古镇建设，持续擦亮文化旅游名片。培育壮大金融主体，确保年底金融业增加值突破100亿元。推进网络经济强市建设。抢抓国家大数据综合试验区建设契机，深入实施“互联网+”行动计划，推进大数据产业园建设。大力发展5G移动通信技术，加快建设北邮国家重点实验室许昌基地、5G泛在小镇，推动5G产业走在全国前列。大力支持实体经济发展。加大“个转企、企升规、规改股、股上市”支持力度，促进企业发展壮大，力争新增规模以上工业企业50家以上、主板上市企业1家、“新三板”挂牌企业3家以上。提升金融服务实体经济的效率和水平，力争新增贷款175亿元、直接融资105亿元。降低实体经济成本，继续清理涉企收费，让企业轻装上阵、降本增效。

（三）强化创新驱动发展。把创新作为第一动力、摆上核心位置，大力实施《许昌市创新驱动发展战略规划（2018-2030）》，加快创新驱动提质增效。发挥政府主导作用。持续加大政府投入，创新体制机制，加快实施51个“双创”示范基地重点项目，扎实创建国家高新区、国家知识产权示范城市，积极融入郑洛新国家自主创新示范区。培育壮大创新主体。强化企业创新主体地位，引导各类技术创新要素向企业集聚，培育引进一批创新引领型企业、人才、平台和机构，新培育高新技术企业30家以上，新建省级创新平台20家以上。深入实施“许昌英才计划”。创新柔性招才引智机制，大力引进高层次创新创业人才和团队，以团队带项目、兴产业。加快高层次人才服务中心和人才公寓建设，开展“一站式”创业服务。提高核心竞争力。引导支持企业加强研发能力建设，深化与中科院、北京邮电大学等大院名校的合作，大力引进转化先进技术成果，推动核心技术自主创新。

（四）着力提升城镇化质量。深入推进以人为核心的新型城镇化，打造城乡区域协调发展新格局。加快推进郑许一体化。坚持立足许昌、对接空港、发挥优势、错位发展，加强产业、生态、交通、功能、平台、空间对接，形成“1+1>2”的协同发展效应。推进产业互补，积极承接郑州航空港、河南自贸区产业辐射和溢出，吸引更多优质产业和企业入驻；加快许港产业带建设，突出抓好许昌中德（欧）产业园和长葛产业新城建设。推进生态共建，打造独具魅力的郑州大都市区“南花园”。推进交通互联，加快郑许市域铁路建设，积极构建郑许间立体快捷交通体系。统筹推进郑许功能重构、平台共享、空间对接。大力实施百城建设提质工程。把百城建设提质与文明城市创建、城市“双修”结合起来，加强城市规划、建设、管理、经营。加快推进395个百城提质项目，确保完成年度投资542亿元。统筹城市地上地下规划建设，科学合理开发利用地下空间。以入选全国老旧小区改造试点城市为契机，加快实施“四改一增”工程。深化中心城区“五城联创”和县（市）城区“三城联创”，巩固创建成果。抓好交通秩序“治乱”、市容卫生“治脏”、生态环境“治污”、公共服务“治差”，让许昌成为创业之都、宜居之城、幸福之家。强化基础能力建设。突出交通先行，加快郑万高铁、郑合高铁、311国道改建等项目建设。持续提升集中供热能力，新增集中供暖5万人。加快水利基础设施建设，推动城乡电网改造提升。建立完善住房制度。坚持“房子是用来住的，不是用来炒的”，加快建立多主体供给、多渠道保障、租购并举的住房制度，促进供求平衡，整治市场秩序，确保房地产市场平稳健康。加快城乡融合发展。深化户籍制度改革，实行进城落户政策“零门槛”，确保新增农业转移人口10万人左右。提升县域治理水平，抓好国家新型城镇化综合试点建设，支持组团城市向心发展，提升神垕、大周等重点示范镇发展水平。

（五）大力实施乡村振兴战略。切实落实农业农村优先发展，按照“产业兴旺、生态宜居、乡风文明、治理有效、生活富裕”的总要求，科学编制实施乡村振兴战略规划，着力推动产业、人才、文化、生态、组织“五大振兴”，加快补齐农业农村短板。大力发展现代农业。加快农业供给侧结构性改革，全面落实“四优四化”，做强“花、菜、药、烟、薯”等优势产业。稳定粮食产能，新建高标准粮田10万亩，发展高效节水灌溉面积15万亩。发展多种形式适度规模经营，新培育新型经营主体1000家以上。持续深化农村改革。加快农村土地制度改革、金融改革、集体产权制度改革，壮大农村集体经济。稳步推进农村房屋不动产登记，探索盘活用好闲置农房和宅基地。建设美丽宜居乡村。落实农村人居环境整治三年行动方案，改善农村基础设施和公共服务，新建改造提升农村公路400公里，新解决农村自来水人口47.2万人。深化与中民投公司合作，加快美丽乡村文化综合体项目建设。加强农村突出环境问题综合治理，深入推进“厕所革命”，逐步解决垃圾、污水处理难题。完善乡村治理体系。加强农村基层组织建设，逐步健全自治、法治、德治相结合的乡村治理体系，让乡村社会充满活力、安定有序。加强农村思想道德建设，不断提高乡村文明程度。

（六）持续深化改革开放。以纪念改革开放40周年为契机，持续抓改革、扩开放，为高质量发展释放动力活力。深化重点领域改革。持续推进“放管服”改革，加快政务服务“一网、一门、一次”改革，大力推进审批服务便民化。深化国企改革，妥善解决债务处理、债权保护、资产处置、职工安置等问题。深入实施质量强市战略，加快建设国家标准化改革创新先行区。稳步推进机构改革，继续深化行政执法、医药卫生、供销社、投融资、财税等体制改革。不断扩大开放招商。积极融入“一带一路”建设，深化对德合作，巩固扩大与欧盟、东盟等地区的交流合作。更加注重招商实效，优化考核体系，强化项目落地，切实提高合同履约率、资金到位率和项目开工率，特别是对富士康、宝藤生物、深圳汇清等重大项目，加强跟踪问效，确保项目落地。鼓励引导“许商”回归创业发展。积极推进开放平台建设，推动河南自贸区许昌联动区尽早获批，加快建设跨境电商查验中心、跨境经贸合作园区和跨境电商基地。促进非公经济健康发展。坚持“政府办好围墙外的事，企业办好围墙内的事”，积极构建“亲”“清”新型政商关系，着力优化营商环境。持续实施企业家培养“十百千”等行动计划，大力弘扬优秀企业家精神，培育造就新型企业家和高素质人才队伍。

（七）加快建设美丽许昌。牢固树立绿水青山就是金山银山理念，认真贯彻中央、省生态环境保护大会精神，像对待生命一样对待生态环境，让经济社会发展更可持续。扎实推进国家生态文明先行示范区建设。坚持生态惠民、生态利民、生态为民，统筹抓好湿地、农田、城市等生态系统建设，积极打造制度创新试验区、静脉产业发展示范区、水生态文明样板区、生态人居典范区。推进生态保护红线、永久基本农田、城镇开发边界三条控制线划定实施。加强国土等自然资源保护和开发，促进各类资源高效利用。加快森林许昌生态建设。以“绿满许昌”行动计划为抓手，以国家储备林基地建设为重点，深入实施国土绿化提速行动，积极推动中华生物园规划编制，加快城郊森林、生态廊道、森林公园、森林乡村建设，增绿量、增容量、增效益，打造生态宜居、充满活力的现代化城市。推动花木产业壮大规模、提档升级，让“中国花木之都”名片更加闪亮。大力发展循环经济。积极发展节能环保产业，开展节能减排技术改造，提高能源清洁化利用水平。加快静脉产业园、大周再生金属循环经济产业园、襄城县煤焦化循环经济产业园建设，支持长葛市建设国家循环经济示范城市。

（八）着力保障和改善民生。顺应人民群众对美好生活的向往，尽力而为，量力而行，腾出更多财力改善民生，让人民群众有更多获得感、幸福感。切实办好民生实事。围绕省定、市定民生实事，紧盯进度，强力推进，确保年底向人民群众兑现承诺。推进教育改革发展。全面贯彻党的教育方针，牢牢把握“九个坚持”，加快推进教育现代化、建设中原教育强市、办好人民满意的教育。深入实施基础教育提升三年攻坚计划，今年再新建改扩建幼儿园33所、中小学65所。增加学前教育资源供给，加快普及高中阶段教育，大力发展职业教育。深化教育改革创新，充分激发教育事业发展生机活力，努力让每个孩子都能享有更加公平而有质量的教育。积极促进就业创业。深入实施全民技能振兴工程，完成职业技能培训10万人次。促进多渠道就业创业，推动农村劳动力转移就业，确保全年新增城镇就业6万人、农村劳动力转移就业4万人，“零就业家庭”动态保持为零。健全社会保障体系。落实全民参保计划，完善城乡居民基本医疗保险、大病保险和困难群众大病补充保险制度，确保户籍人口应保尽保。健全农村留守儿童和妇女、老年人、残疾人关爱服务体系，加强社会救助、社会福利、优抚安置工作，让每一个身处困境者都能得到社会关爱和温暖。加快健康许昌建设。深化公立医院综合改革，完善分级诊疗制度。加快市中心医院新院区、市妇幼保健院迁建等项目建设。提升基层医疗卫生服务能力，实现中心城区社区卫生服务中心全覆盖。发展老龄事业和产业，推进医养结合，建设国家中医药综合改革试验区。深入推进“双安双创”，扎实创建国家食品安全示范城市，让人民群众吃得更安全、更放心。推动文化体育事业发展。深入实施文化惠民工程，完善公共文化服务功能，加快基层综合文化服务中心等项目建设，积极创建国家公共文化服务示范区。推动文化产业发展，激发文化创新创造活力。广泛开展全民健身活动，办好市第八届运动会。

加强国防教育、国防动员和国防后备力量建设，支持驻许解放军、武警部队、预备役部队、民兵和人民防空建设，深入开展双拥共建，巩固军政军民团结。支持工会、共青团、妇联等人民团体更好发挥桥梁纽带作用。做好民族、宗教、外事、侨务、港澳、对台工作。加强统计、史志、档案、气象、地震、邮政、援疆等工作。

三、提升政府治理能力

顺应人民新期待，开启发展新征程，必须坚定不移加强政府自身建设，推动职能作风新转变、治理能力新提升，建设人民满意政府。

（一）讲政治，树牢“四个意识”。坚持以政治建设为统领，牢固树立“四个意识”，坚定“四个自信”，坚决维护习近平总书记的核心地位，坚决维护党中央权威和集中统一领导，紧跟总书记步伐，紧扣党中央节拍，做到思想上高度认同、政治上坚决维护、组织上自觉服从、行动上紧紧跟随，始终同以习近平同志为核心的党中央保持高度一致。

（二）讲法治，深化依法行政。严格遵守宪法法律，加快法治政府建设，把政府活动全面纳入法治轨道。落实行政执法责任制，严格规范公正文明执法，有权不任性，用权受监督。认真办理人大代表建议和政协委员提案，自觉接受人大法律监督、工作监督和政协民主监督，主动接受社会和舆论监督，认真听取各民主党派、工商联、无党派人士和各人民团体意见。

（三）讲宗旨，恪守为民情怀。始终坚持以人民为中心的发展思想，扎实开展“不忘初心、牢记使命”主题教育，全心全意服务人民。推进政务公开、信息公开，畅通民意诉求渠道。深化“互联网+政务服务”，让企业和群众办事像“网购”一样方便。努力把群众所有“烦心事”“操心事”都一件一件办好，让人民共享美好生活。

（四）讲担当，持续改进作风。大力弘扬焦裕禄同志对群众的那股亲劲、抓工作的那股韧劲、干事业的那股拼劲，紧贴新时代历史任务，紧贴中原更加出彩使命，全面提升“八种本领”，干出实打实的新业绩。坚持求真务实，讲真话、出实招、做实事、求实效，一锤一锤钉钉子，一项一项抓落实。持之以恒纠正“四风”，大力整治变异的形式主义、官僚主义问题，从严整治不作为、慢作为、乱作为。

（五）讲廉洁，坚守廉政底线。认真履行全面从严治党主体责任和“一岗双责”，严明政治纪律和政治规矩，纵深推进政府系统党风廉政建设和反腐败斗争。严格落实中央八项规定及实施细则精神，坚决从严查处各类腐败问题和侵害群众利益行为，保持惩治腐败高压态势！

各位代表！

新征程任重道远，新使命催人奋进。让我们更加紧密地团结在以习近平同志为核心的党中央周围，高举习近平新时代中国特色社会主义思想伟大旗帜，在省委、省政府和市委的坚强领导下，不忘初心，牢记使命，锐意进取，奋勇争先，为许昌在谱写新时代中原更加出彩新篇章中走在前列而不懈奋斗
